# Intrauterine Inflammation Alters the Transcriptome and Metabolome in Placenta

**DOI:** 10.3389/fphys.2020.592689

**Published:** 2020-11-05

**Authors:** Yu-Chin Lien, Zhe Zhang, Guillermo Barila, Amy Green-Brown, Michal A. Elovitz, Rebecca A. Simmons

**Affiliations:** ^1^Department of Obstetrics and Gynecology, Maternal and Child Health Research Center, Perelman School of Medicine, University of Pennsylvania, Philadelphia, PA, United States; ^2^Department of Pediatrics, Children’s Hospital of Philadelphia, Philadelphia, PA, United States; ^3^Center for Biomedical Informatics, Children’s Hospital of Philadelphia, Philadelphia, PA, United States

**Keywords:** placenta, transcriptome, metabolome, inflammation, spontaneous preterm birth, bioenergetic metabolism

## Abstract

Placental insufficiency is implicated in spontaneous preterm birth (SPTB) associated with intrauterine inflammation. We hypothesized that intrauterine inflammation leads to deficits in the capacity of the placenta to maintain bioenergetic and metabolic stability during pregnancy ultimately resulting in SPTB. Using a mouse model of intrauterine inflammation that leads to preterm delivery, we performed RNA-seq and metabolomics studies to assess how intrauterine inflammation alters gene expression and/or modulates metabolite production and abundance in the placenta. 1871 differentially expressed genes were identified in LPS-exposed placenta. Among them, 1,149 and 722 transcripts were increased and decreased, respectively. Ingenuity pathway analysis showed alterations in genes and canonical pathways critical for regulating oxidative stress, mitochondrial function, metabolisms of glucose and lipids, and vascular reactivity in LPS-exposed placenta. Many upstream regulators and master regulators important for nutrient-sensing and mitochondrial function were also altered in inflammation exposed placentae, including STAT1, HIF1α, mTOR, AMPK, and PPARα. Comprehensive quantification of metabolites demonstrated significant alterations in the glucose utilization, metabolisms of branched-chain amino acids, lipids, purine and pyrimidine, as well as carbon flow in TCA cycle in LPS-exposed placenta compared to control placenta. The transcriptome and metabolome were also integrated to assess the interactions of altered genes and metabolites. Collectively, significant and biologically relevant alterations in the placenta transcriptome and metabolome were identified in placentae exposed to intrauterine inflammation. Altered mitochondrial function and energy metabolism may underline the mechanisms of inflammation-induced placental dysfunction.

## Introduction

Preterm birth (delivery before or at 37 weeks of gestation) is the leading cause of neonatal morbidity and mortality worldwide (March of Dimes, 2015). 15 million babies are born prematurely annually resulting in an excess of 1 million deaths. It is estimated that premature births cost the American healthcare system at least $26 billion a year (Institute of Medicine, 2007). Spontaneous preterm birth (SPTB) remains a significant and poorly understood perinatal complication. SPTB includes preterm spontaneous rupture of membranes, cervical weakness, and preterm labor. While the exact etiology of SPTB remains unknown, many factors may contribute, including placental dysfunction, cervical insufficiency, uterine distension, vascular disorders, and chorioamnionitis (Romero et al., 2014; Manuck et al., 2015).

Infection is detected in at least 25% of all preterm birth cases (Simmons et al., 2010; Thaxton et al., 2010). It has been reported that up to 70% of patients with extreme SPTB test positive for an infectious agent, suggesting that intrauterine infection and infection-associated inflammation play a critical role in SPTB (Watts et al., 1992; Onderdonk et al., 2008). How intrauterine infection/inflammation precisely induces parturition is unknown, however, emerging evidence supports the concept that preterm births complicated by chorioamnionitis are associated with placental insufficiency (Kourtis et al., 2014; Morgan, 2016; do Imperio et al., 2018). Therefore, elucidating underlying mechanisms for placental dysfunction may lead to a better understanding of the etiology of prematurity and the development of preventative treatment.

During pregnancy, the placenta facilitates nutrient transport and gas exchange, and supports growth and development of the fetus. It also produces and releases hormones into maternal and fetal circulation to regulate uterine function, maternal metabolisms, fetal growth and development. The placenta produces a wide variety of metabolites, and many of which are involved in energy production (Battaglia, 1992; Shekhawat et al., 2003). The placenta also protects the fetus against infections, toxins, xenobiotic molecules, and maternal diseases (Sultana et al., 2017). Therefore, a well-functioning placenta is crucial for normal gestation. We hypothesized that intrauterine inflammation leads to deficits in the capacity of the placenta to maintain bioenergetic and metabolic stability throughout the course of pregnancy ultimately resulting in SPTB.

To test this hypothesis, we assessed the transcriptome and metabolome in our well-established mouse model of intrauterine inflammation that leads to preterm delivery (Elovitz and Mrinalini, 2005). As infection that precipitates preterm birth is an acute process, the aim of our study was to identify novel pathways in the placenta that are altered in the setting of acute intrauterine inflammation that will provide insight into the underlying mechanisms driving SPTB.

## Materials and Methods

### Intrauterine Inflammation Animal Model

The University of Pennsylvania Institutional Animal Care and Use Committee approved all experiments in this study. Details of the intrauterine inflammation model have been previously published (Elovitz and Mrinalini, 2005; Elovitz et al., 2011; Hester et al., 2018; Brown et al., 2019). CD-1 out-bred, timed pregnant mice were purchased from Charles River Laboratories (Wilmington, MA). Briefly, a mini-laparotomy was performed under isoflurane anesthesia on CD-1 timed pregnant mice at gestational day 17 (E17), with normal gestation being 19 days. The right lower uterus was exposed allowing visualization of the lower two gestational sacs. Mice then received intrauterine injections of liposaccharide (LPS) from Escherichia coli (055:B5, Sigma, St. Louis, MO, L2880, 50 μg/100 μl phosphate buffered saline/animal) or PBS (100 μl/animal) (LPS, *n* = 4; PBS, *n* = 4) between two gestational sacs. Surgical incisions were closed using surgical staples and dams were allowed to recover for 6 h prior to placental tissue collection. LPS dose of 50 μg per animal does not result in maternal mortality and is the lowest dose that can induce preterm labor. The number of fetuses in each dam ranged from 11 to 16. Four placentas from each dam (two placentas from each side of injection point) were collected and flash frozen in liquid nitrogen.

### Total RNA Isolation and RNA-Seq Library Preparation

Total RNA was extracted from frozen placenta of gestational day 17 pregnant animals using TRIzol^®^ Reagent (Invitrogen), followed by Qiagen RNeasy^®^ Mini Columns following manufacturer’s instructions. RNA integrity numbers greater than 7 were used for RNA-Seq Studies. Real-time q-PCR was used to determine the sex of the placenta using TaqMan probes for Xist as a positive control (Mm01232884_m1) and Sry (Mm00441712_s1) as a marker of male sex (Applied Biosystems). Four RNA-Seq libraries for each gender and experimental group were generated using the Illumina TruSeq Stranded Total RNA Sample Prep Kit with Ribo-Zero by Beijing Genomics Institute (BGI) at the Children’s Hospital of Philadelphia.

### RNA-Seq and Gene Expression Analysis

RNA-Seq Libraries were paired-end sequenced to 100 bp on an Illumina HiSeq platform at either Beijing Genomics Institute (BGI) at the Children’s Hospital of Philadelphia or Next-Generation Sequencing Core at The University of Pennsylvania. RNA-seq data in.fastq files were aligned to reference mouse genome (mm10) and transcriptome using (STAR)^1^ (Spliced Transcripts Alignment to a Reference) program. STAR was run in the 2-pass mode. The first pass mapped reads to known splice junctions in reference transcriptome during alignment while allowing for detection of novel junction sites. The second pass re-aligned reads to the reference genome and transcriptome, plus novel junction sites detected by the first pass. The alignment results were saved as indexed.bam files. Aligned reads in.bam files were loaded into R and mapped to known exons, transcripts, and genes. Read pairs uniquely mapped to the sense strand of known genes were count to obtain a gene-level read count. Read counts were converted to FPKM (fragment per kilobase per million reads) to represent gene expression level. One control sample was considered as an outlier according to the principal component analysis. This sample accounted for 39% of total variance in the control samples and had abnormally high expression of liver-specific proteins, such as albumin and apolipoproteins, suggesting a contamination with liver cells. Therefore, this sample was excluded from the analysis of differential gene expression. Differential gene expression between the LPS- and saline-treated groups was tested by the DESeq2 method. Differentially expressed genes were selected based on their fold changes, DESeq2 *p*-values and corresponding false discovery rate. Sequence data have been deposited in NCBI’s Gene Expression Omnibus and are accessible through GEO Series accession number GSE151728. Functional analysis was conducted using QIAGEN’s Ingenuity^®^ Pathway Analysis (IPA^®^). Core analyses were performed on genes with FDR (*q*-value) < 0.05.

### Metabolomics

Primary metabolomic analysis was performed on the same placenta tissue used for RNA-Seq (*n* = 8 in each experimental group). Placental samples were collected, flash frozen, and stored at −80°C prior to metabolite extraction and analysis at Metabolon (Durham, NC, United States). The sample preparation process was carried out using the automated MicroLab STAR^®^ system from Hamilton Company. The resulting extract was divided into two fractions; one for analysis by LC/MS/MS and one for analysis by GC/MS. Biochemicals were analyzed in four ways: (1) acidic positive ion conditions (water and methanol), optimized for hydrophilic compounds; (2) acidic positive ion conditions (water, methanol and acetonitrile), optimized for hydrophobic compounds; (3) basic negative ion conditions; (4) negative ionization. Samples were placed briefly on a TurboVap^®^ (Zymark) to remove the organic solvent. Each sample was then frozen and dried under vacuum. Analytes were extracted and prepared using Metabolon’s standard solvent extraction method (DeHaven et al., 2012; Evans et al., 2014). The extracted samples were split into equal parts for analysis on complementary GC/MS (gas chromatography mass spectrometry) and LC/MS (liquid chromatography mass spectrometry) platforms.

The LC/MS portion of the platform was based on a Waters ACQUITY UPLC and a Thermo-Finnigan LTQ-FT mass spectrometer, which had a linear ion-trap (LIT) front end and a Fourier transform ion cyclotron resonance (FT-ICR) mass spectrometer. For ions with signals greater than 2 million, accurate mass measurement was performed. Accurate mass measurements were made on the parent ion as well as fragments. The typical mass error was less than 5 ppm. Ions with less than two million signals required additional efforts to characterize. Fragmentation spectra (MS/MS) were typically generated, but when necessary, targeted MS/MS was employed.

The samples for GC/MS analysis were re-dried under vacuum desiccation for a minimum of 24 h prior to being derivatized under dried nitrogen using N,O-bis(trimethylsilyl)-flouroacetamide (BSTFA). The GC column was 5% phenyl and the temperature ramp ranged from 40° to 300° C in a 16 min period. Samples were analyzed on a Thermo-Finnigan Trace DSQ fast-scanning single-quadrupole mass spectrometer using electron impact ionization. Compounds were identified by comparison to Metabolon library entries of purified standards or recurrent unknown entities.

### Integrated Network Analysis of the Transcriptome and Metabolome

Differentially expressed genes and metabolites whose levels were significantly altered were analyzed using the MetScape3.1 (Karnovsky et al., 2012) in Cytoscape (v3.8.0). Mouse genes were mapped to their corresponding human homologs, and the interactome networks were generated based on known protein-protein and protein-metabolite interactions using human data. The metabolic pathways which were associated with protein-metabolite interactions were mapped onto each network.

## Results

### Intrauterine Inflammation Alters Transcriptome Profiles in Placenta

**FIGURE 1:**
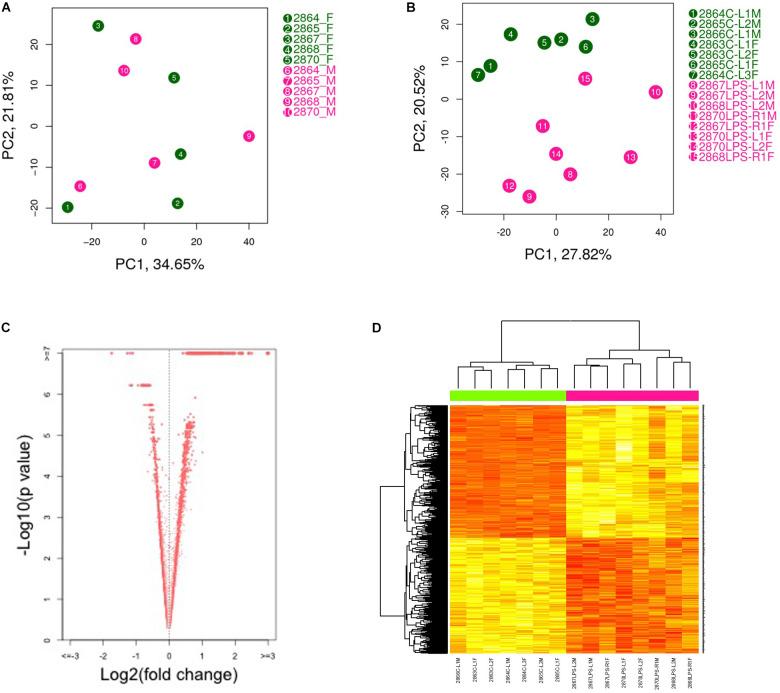
Principal component analysis (PCA plot) of placental transcriptomes and differentially expressed genes. **(A)** PCA plots showed a strong impact of the litter but no significant differences between two genders. **(B)** PCA plot revealed significant separation between saline- and LPS-exposed groups. **(C)** Volcano plots identifying differentially expressed genes with an FDR (*q*-value) < 0.05. **(D)** Heat map of differentially expressed genes showing significant differences between saline- and LPS-exposed groups. Each row in the heat map corresponds to data point from a single gene, whereas columns correspond to individual samples. The branching dendrogram corresponds to the relationships among samples, as determined by clustering using differentially expressed genes. Up- and down-regulation of gene expressions are shown on a continuum from yellow to red, respectively. *N* = 7 for saline group and *N* = 8 for LPS group.

Principal component analysis (PCA) showed a strong confounding impact of treatment but not of fetal sex (Figure 1A). This suggests that LPS-induced transcriptome changes in placenta are not affected by sex hormones or sex chromosomes at this gestational age. As expected, the two treatment groups were readily distinguishable (Figure 1B). Thus, we combined the data generated from male and female placentas from the same dam for further analysis. Using an FDR (*q*-value) ≤ 0.05, 1871 transcripts were differentially expressed in placenta from LPS-exposed dam. Among them, 1149 transcripts were up-regulated and 722 transcripts were down-regulated (Figures 1C,D and Supplementary Table S1).

Ingenuity pathway analysis (IPA) revealed over 200 canonical pathways that were altered by intrauterine LPS treatment. As expected, many of these pathways were related to immune system changes, including: granulocyte/agranulocyte adhesion and diapedesis; communication between innate and adaptive immune cells; B cell/T-cell/dendritic cell maturation, differentiation, and activation; Th1 pathway; Th2 pathway; death receptor signaling; as well as many pro- and anti-inflammatory cytokine-related signaling pathways (Supplementary Table S2). Beyond these alterations in immune system pathways that are likely to be directly due to LPS-induced intrauterine inflammation, many additional pathways were altered in LPS-exposed animals. As predicted by activation z-score, top pathways activated in LPS-exposed animals included renin-angiotensin signaling, p38 MAPK signaling, insulin resistance, retinoic acid mediated apoptosis signaling, PDGF signaling, NF-κB signaling, ceramide signaling, JAK/Stat signaling, PI3K/AKT signaling and sphingosine-1-phosphate signaling (Table 1). The top pathways inhibited in LPS-exposed animals included PPAR signaling, PPARα/RXRα activation, antioxidant action of vitamin C, PTEN signaling, and STAT3 pathway (Table 1).

**TABLE 1:** Top ingenuity canonical pathways altered by intrauterine inflammation in placenta.

Pathways	*p*-value	Activation z-score
Renin-angiotensin signaling	3.02E-04	3.77
P38 MAPK signaling	1.55E-07	3.40
Insulin resistance signaling	9.33E-06	3.30
Retinoic ACID MEDIATED APOPTOSIS SIGNALING	7.59E-05	2.89
PDGF signaling	1.66E-03	2.84
NF-κB signaling	1.26E-12	2.65
Ceramide signaling	2.51E-05	2.18
JAK/Stat signaling	4.47E-06	2.06
PI3K/AKT signaling	6.03E-05	1.53
Sphingosine-1-phosphate signaling	1.38E-02	1.50
PPAR signaling	4.47E-09	−3.40
PPARα/RXRα activation	3.98E-03	−1.50
Antioxidant action of vitamin C	3.31E-04	−3.00
PTEN signalling	7.24E-04	−1.89
STAT3 pathway	5.62E-04	−1.07

#### Vascular Reactivity Is Altered in Response to Intrauterine Inflammation

Intrauterine inflammation altered multiple pathways that modulate vascular biology (Supplementary Table S3) including the renin-angiotensin signaling pathway which was highly activated (z-score = 3.77). Twenty genes comprising this pathway were differentially expressed, including *Ccl2, Ccl5, Stat1, Tnf, Pik3r5, Nfkb1*, and *Nfkb2* (Supplementary Table S3). Other signaling pathways related to vascular function are listed in Supplementary Table S3 and include platelet-derived growth factor (PDGF), P2Y purigenic receptor, sphingosine-1 phosphate, PI3K/AKT, and PTEN. PDGF is important in the pathogenesis of pregnancy-induced hypertension and is thought to regulate placenta vascular remodeling (Morita et al., 2001; Hoch and Soriano, 2003). This pathway was predicted to be activated (z-score = 2.84) in LPS-exposed placenta, and included genes such as *Stat1, Eif2ak2*, and *Inpp5b*. P2Y purigenic receptor signaling pathway, which mediates purine and pyrimidine signaling, was also activated (z-score = 2.84) (Motte et al., 1995). Genes in this pathway modulate endothelial production of vasodilator prostacyclin (PGI2) and nitric oxide (NO). *P2ry2, Prkch, Myc*, and *Plcd3* were a few examples of genes whose expression was altered in this pathway.

#### Intrauterine Inflammation Increases Oxidative Stress and Mitochondrial Dysfunction

In previous studies, we and others have reported that mitochondrial dysfunction and other metabolic abnormalities in the placenta are linked to SPTB in the human (Zhang et al., 2010; Crawford et al., 2018; Martin et al., 2018; Elshenawy et al., 2020). Consistent with these previous findings, IPA canonical pathway analysis revealed that intrauterine LPS treatment was associated with dysregulation of redox status/increased oxidative stress, and mitochondrial dysfunction in placenta (Table 1 and Supplementary Table S4). IPA can also predict altered downstream cellular processes and biological functions. Using this “diseases and biological functions” analysis, more than 130 genes differentially expressed in LPS-exposed animals were identified that play a role in free radical synthesis and metabolism, which were predicted to be increased in LPS-exposed placenta (*p* = 1.76E-28 ∼ 1.54E-14, Supplementary Table S5). IPA-Tox analysis uses “toxicity functions” in combination with “toxicity lists” to link gene expression to clinical pathology endpoints. Consistent with our canonical pathway analysis, IPA-Tox analysis further identified genes directly associated with mitochondrial dysfunction, such as *Il1b, Tnf, Lcn2, Cd40, Pawr*, and *Timp3* (Supplementary Table S6). Expression changes in these genes have been shown to alter mitochondrial transmembrane potential, increase depolarization of mitochondria, increase damage of mitochondria, and increase oxidative stress. Finally, intrauterine inflammation appears to impair free-radical scavenging in the placenta. Vitamin C, a primary antioxidant, can quench ROS directly, and its oxidized form, dehydroascorbic acid, inhibits IKK, and NF-κB mediated signal transduction (Cárcamo et al., 2004). Interestingly, the antioxidant action of vitamin C was predicted to be inhibited in LPS-exposed animals (*z*-score = −3.0) which is consistent with activation of NF-κB. *Txnrd1, Plcd1*, and *Pla2g7* were a few examples of genes whose expression was changed in this pathway (Supplementary Table S4).

Elevated levels of ROS can activate the NF-κB pathway which stimulates the innate immune response and results in production of cytokines that are detected in amniotic fluid samples preceding spontaneous preterm delivery (Zhang et al., 2010). Consistent with our findings of increased oxidative stress and mitochondrial dysfunction, NF-κB signaling was activated in LPS-exposed animals (z-score = 2.65). Forty-three genes comprising this pathway were differentially expressed (Supplementary Table S3). Among them, 36 genes in this pathway, including *Eif2ak2, Il1b, Cd40, Il1rn, Tlr2, Tnfaip3*, and *Il1a* were up-regulated (Supplementary Table S4).

#### Intrauterine Inflammation Alters Glucose and Lipid Metabolic Pathways

Peroxisome proliferator-activated receptor (PPAR) signaling and PPARα/RXRα activation play a central role in regulating fatty acid oxidation and lipid/cholesterol metabolism, as well as maintain glucose and amino acid homeostasis (Zelcer and Tontonoz, 2006; Feldman et al., 2008; Tyagi et al., 2011; Hong and Tontonoz, 2014). Both pathways were both predicted to be inhibited (z-score = −3.40 and −1.50, respectively). Thirty-seven genes comprising these pathways were differentially expressed in LPS-exposed animals (Supplementary Table S7). The PPAR family of transcription factors PPARs also exert anti-oxidant effects, and are critically important to placental function (Stienstra et al., 2007; Matsuda et al., 2013). Consistent with our finding of altered PPAR signaling, fatty acid synthesis and metabolism were predicted to be increased as well. More than 180 differentially expressed genes were identified that regulate fatty acid synthesis, transport, and metabolism (*p* = 8.94E-25∼1.35E-18), such as *Apoa1, Apoa2, Hnf4α, Lpl*, and *Fabp6* (Supplementary Table S5).

Additional lipid signaling pathways that were also altered including ceramide and sphingosine-1-phosphate signaling which were both predicted to be activated in LPS-exposed animals (z-score = 2.18 and 1.50, respectively). A total of 29 genes comprising these pathways were differentially expressed, including *Pik3r5, Pik3r1, Pik3cd, Smpd1, S1pr3*, and *S1pr1* (Supplementary Table S8). Ceramides can modulate mitochondrial function and oxidative phosphorylation (Kogot-Levin and Saada, 2014) and are major regulators of vascular integrity (Sattler and Levkau, 2009).

Glucose metabolism plays a fundamental role in placenta function. 293 differentially expressed genes were identified to regulate glucose metabolism (*p* = 3.13E-39), including *Il6, Stat3, Ucp3, Nr4a1, Tgfb1, Ins2*, and *Irs3* (Supplementary Table S5). Many of these genes are involved in insulin signaling which was predicted to be activated (z-score = 3.30) by intrauterine inflammation in placenta. Genes that were dysregulated in this pathway include *Irs3, Ins2, Socs1, Socs2*, and *Socs3* (Supplementary Table S8). Other insulin signaling pathways including PI3K/AKT were predicted to be activated in LPS-exposed animals (z-score = 1.53). Consistent with this finding, the inhibitor PTEN (phosphatase and tensin homolog) signaling, was inhibited (z-score = −1.89) (Supplementary Table S3). Twenty-nine genes comprising these pathways were differentially expressed, such as *Nfkß1, Nfkß2, Inpp5b, Cdkn1α*, and *Pik3cd*. These pathways regulate glucose and lipid metabolism and oxidative stress through modulating mitochondrial function (Bijur and Jope, 2003; Cheng et al., 2010; Goo et al., 2012). Via PI3K signaling, the placenta can fine-tune the supply of maternal nutrient resources to the fetus (Sferruzzi-Perri et al., 2016).

While the precise role of p38MAPK in the placenta is unclear, there are reports that activation of p38MAPK signaling is associated with premature rupture of membranes and subsequent preterm birth (Menon, 2016). This pathway was markedly altered by intrauterine inflammation and 26 genes in the p38 MAPK signaling pathway were differentially expressed leading to predicted activation by intrauterine LPS treatment (z-score = 3.40) (Supplementary Table S9). This pathway is triggered by stress stimuli such as oxidative stress, inflammatory cytokines, and death ligands (Cuenda and Rousseau, 2007).

#### Intrauterine Inflammation Alters Upstream Regulators and Regulatory Networks Important for Nutrient-Sensing and Mitochondrial Function

**FIGURE 2:**
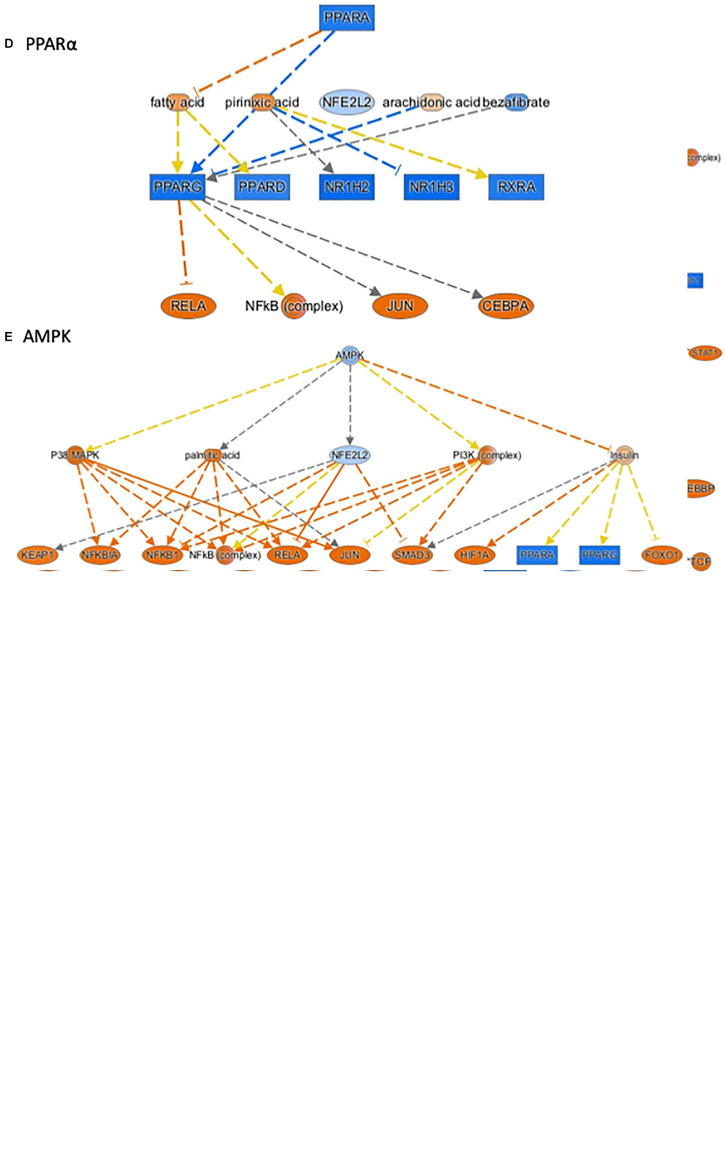
Ingenuity Pathway Analysis^®^ (IPA) annotated mechanistic networks regulated by critical upstream regulators. Differentially expressed genes regulated by FOXO1 **(A)**, mTOR **(B)**, HIF1A **(C)**, PPARα **(D)**, and AMPK **(E)**. Orange-filled and blue-filled shapes indicate predicted activation and inhibition, respectively, red-filled and green-filled shapes indicate increased and decreased expression, respectively, orange-red lines indicate activation; blue lines indicate inhibition; yellow lines indicate findings inconsistent with state of downstream activity; gray lines indicate that the effect was not predicted.

Ingenuity pathway analysis can identify upstream transcriptional regulators that can explain the changes in gene expression and biological activities. In addition to LPS and the pro-inflammatory cytokines, such as TNF, IL1ß, and IFNγ, which may be induced in response to LPS treatment, we identified several upstream transcriptional regulators that are critical for nutrient-sensing and mitochondrial function in the placenta. The top activated upstream regulators included NFκB, STAT1, FOXO1, FOXO4, HIF1A, mTOR, the glucose sensor-GSK3, and APP (Table 2 and Figures 2A–D). STAT1 is a transcription activator and also localizes to the mitochondria and regulates mitochondrial biogenesis and mitochondrial encoded transcripts (Meier and Larner, 2014). FOXO1 and FOXO4 are two members of FOXO (forkhead box protein O) transcription factors and play crucial roles in regulating glucose metabolism, adipogenesis, energy homeostasis, oxidative stress, and mitochondrial function (Nakae et al., 2001, 2003; Carter and Brunet, 2007; Daitoku and Fukamizu, 2007; Kim and Koh, 2017). mTOR (mammalian target of rapamycin) is a central coordinator of metabolism and regulates energy-sensing pathways partly through regulating mitochondrial function and AMPK activity (Tokunaga et al., 2004; Kennedy and Lamming, 2016). APP (amyloid β-precursor protein), a transmembrane glycoprotein. Interestingly, the human placenta abundantly expresses APP and APP-processing enzymes, which are up-regulated in preeclampsia (Buhimschi et al., 2014). β-Amyloid aggregates are also present in placentas of women with preeclampsia and fetal growth restriction. Important upstream regulators that were inhibited in placentas from LPS-exposed dams included PPARα (peroxisome proliferator-activated receptor alpha), AMPK (AMP-activated protein kinase), TRIM24 (tripartite motif containing 24), PTGER4 (prostaglandin E receptor 4), NKX2-3, and ACKR2 (atypical chemokine receptor 2) (Table 2 and Figure 2E). PPARα plays a major role in lipid metabolism/homeostasis regulation (Kersten et al., 1999). It also involves in embryonic and placental development and differentiation in response to nutritional stimuli (Matsuda et al., 2013). AMPK is a master metabolic regulator controlling glucose sensing and uptake, lipid metabolism, glycogen, cholesterol and protein synthesis, and induction of mitochondrial biogenesis (Winder and Hardie, 1999; Claret et al., 2007). Placental AMPK is important in regulating nutrient transport (Carey et al., 2014). AMPK also modulates acute inflammatory reactions and pro-labor mediators (Lim et al., 2015). PTGER4 is a receptor for prostaglandin E2 (PGE2), a natural prostaglandin and a medicine commonly used in labor induction. PTGER4 also regulates lipid metabolism (Cai et al., 2015). Placenta is the richest source of ACKR2, which acts as a chemokine scavenger (Madigan et al., 2010). It has been shown that Ackr2 deficiency in mice leads to abnormal placenta structure, increases the incidence of stillbirth, and reduces neonatal survival (Teoh et al., 2014).

**TABLE 2:** Top upstream regulators altered by intrauterine inflammation in placenta.

Regulators	*p*-value	Activation z-score	# Genes regulated
NFkB (complex)	1.97E-46	9.74	158
STAT1	1.41E-67	7.81	130
APP	1.57E-38	7.67	180
OSM	3.04E-31	5.93	124
FOXO1	3.07E-12	5.65	65
HIF1A	3.76E-14	4.09	74
ETS1	5.00E-08	3.22	40
SP1	7.31E-18	3.14	101
ELK1	1.46E-05	2.39	14
FOXO4	1.60E-05	2.22	13
mTOR	2.25E-06	1.40	43
GSK3	3.69E-05	0.57	16
TRIM24	4.02E-47	−7.26	63
PTGER4	6.47E-45	−6.46	73
NKX2-3	3.26E-24	−5.22	66
ACKR2	7.21E-28	−5.11	30
PPARA	7.49E-11	−1.58	71
AMPK	2.05E-05	−0.58	22

The causal network analysis in IPA can expand predictions to identify potential novel master-regulators responsible for the changes in gene expression. Not surprisingly, the top hits for activated master regulators were regulators of the immune system, including toll-like receptors (TLRs), interferon regulatory factor 3 (IRF3), interferon receptors, and interferon induced with helicase C domain 1 (IFIH1) (Supplementary Table S10). We also identified several additional interesting master regulators that were altered in LPS-exposed placenta (Table 3 and Supplementary Figure S1). TANK binding kinase 1 (TBK1) was highly activated with a z-score of 12.15. TBK1 is similar to IKB kinases and can mediate NF-κB activation (Pomerantz and Baltimore, 1999), which was consistent with our pathway analysis that NF-κB signaling was activated in LPS-exposed animals (z-score = 2.65). TBK1 modulated activities of 20 regulators, including NFκB complex, IKK complex, AKT, IRFs, JUN, and STAT1. MAVS (mitochondrial antiviral signaling protein), which was predicted to be activated, is a membrane protein found in the outer mitochondrial membrane, peroxisomal membrane, and the mitochondrial associated membrane of the endoplasmic reticulum (Dixit et al., 2010; Horner et al., 2011). Fifteen regulators were modulated by MAVS, including AP1, ATF2, CASP1, JNK, and NFκB complex. STIM1 was predicted as an activated master regulator (*z* = 9.85) and modulated 8 regulators in our dataset. STIM1 plays a crucial role in coordinating calcium signals, oxidative stress, and regulating mitochondrial shape and bioenergetics (Henke et al., 2012; Soboloff et al., 2012). Both STAT3 and STAT1 were predicted as activated master regulators. STAT3, a transcription activator, is also localized in the mitochondria and critical for the optimal activities of Complexes I and II of electron transport chain (Wegrzyn et al., 2009). STAT3 also regulates mitochondrial membrane potential, mitochondrial permeability transition pore, ROS, and ATP production (Meier and Larner, 2014). In addition to modulating four common regulators, CASP9, ERK1/2, JUN, and MAPK, STAT3 and STAT1 modulated activities of another 18 and 16 regulators, respectively. RNF216 and TRIM21 were two master regulators predicted to be inhibited. They modulate activities of 6 and 9 regulators, respectively. RNF216 (ring finger protein 216) inhibits TNF- and IL1-induced NF-kB activation pathway, as well as functions as an E3 ubiquitin-protein ligase (Miah et al., 2011). TRIM21 (tripartite motif containing 21) is also an E3 ubiquitin-protein ligase and negatively regulates the innate immune response (Higgs et al., 2008). Trim21 can regulate de novo lipogenesis by interacting with acetylated fatty acid synthase (FASN) and promoting FASN polyubiquitylation and destabilization (Lin et al., 2016).

**TABLE 3:** Top master regulators altered by intrauterine inflammation in placenta.

Master regulators	*p*-value	Activation z-score	# connected regulators	Connected regulators
TBK1	5.33E-77	12.94	20	Akt, AKT1, ATF2, I kappa b kinase, Ikb, IKBKB, IKK (complex), IRF3, IRF5, IRF7, JUN, NFkB (complex), NFKBIA, REL, RELA, SQSTM1, STAT1, TANK, XIAP, ZBP1
MAVS	8.97E-67	12.27	15	Ap1, ATF2, CASP1, I kappa b kinase, IFNB1, IKBKE, IRF3, IRF7, Jnk, JUN, NFkB (complex), PYCARD, RELA, TBK1, TRAF3
STIM1	4.95E-55	10.64	8	EIF2AK3, Nfat (family), NFATC2, NFkB (complex), ORAI1, PRKAA, TRPC1, voltage-gated calcium channel
STAT3	2.76E-64	3.78	22	Akt, AR, CASP8, CASP9, CEBPA, CHUK, CTNNB1, ERK, ERK1/2, FOS, GC-GCR dimer, GSK3B, JAK2, JUN, Mapk, MET, mir-21, PI3K (complex), RHOA, SRC, STAT1, TP63
STAT1	1.13E-72	1.96	20	BCL2L1, CASP1, CASP3, CASP9, CCL2, CDK2, CXCL10, CYP2E1, EIF2AK2, ERK1/2, Ifn, JUN, MAPK1, NFkB (complex), NOS2, RUNX2, SMAD3, STAT3, STAT5a/b, TBX21
RNF216	6.26E-78	−12.43	6	IRF3, NFkB (complex), RIPK1, TLR4, TLR9, TRAF3
TRIM21	1.64E-79	−12.01	9	CDKN1B, IKBKB, IRF3, IRF7, IRF8, NFkB (complex), TRAF6, TRIM5, USP4

Taken together, our RNA-seq data demonstrate that intrauterine inflammation causes marked abnormalities in mitochondria function, metabolism, vascular reactivity and key pathways regulating placenta development.

### Intrauterine Inflammation Alters the Metabolome in Placenta

#### Global Assessment of Metabolomics Data

**FIGURE 3:**
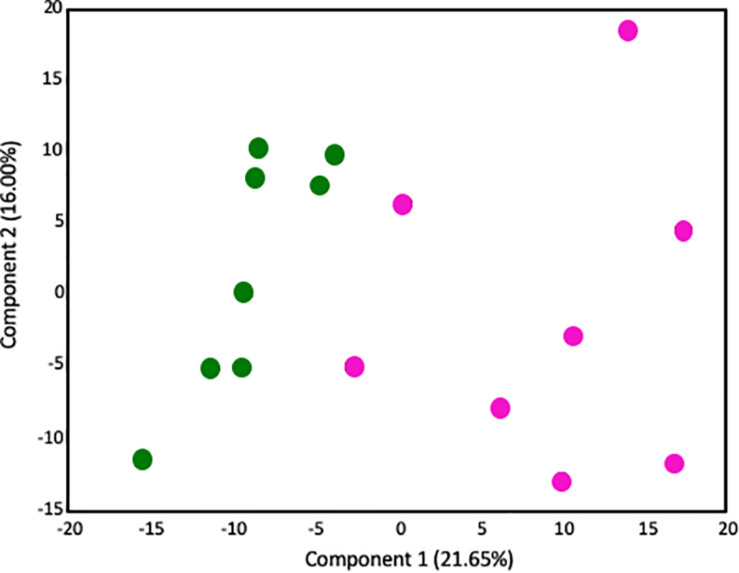
Principal components analysis demonstrates that the global metabolome from LPS-exposed placenta samples is overall distinguishable from the metabolome of control placenta samples. Control placentas: green circles (*N* = 8); LPS-exposed placenta: pink circles (*N* = 8). The *X*-axis (Comp1) represents 21.65% of the variability between samples and the *Y*-axis (Comp 2) represents 16.00% of the variability between samples.

Given that our transcriptome results implicated significant changes in metabolism in the placenta, we next performed metabolic profiling in placenta to investigate whether changes in the transcriptome correlated with changes in the metabolome. There were no sex-specific effects. 547 biochemical compounds were detected in the placentas studied, of which 57 were significantly increased and 93 were significantly decreased in LPS-exposed placenta (a *p*-value < 0.05 and *q*-value < 0.1 was considered significant). Principal component analysis (PCA) identified a shift in the global metabolic profile in LPS placenta, indicating that the two groups were strongly distinguishable (Figure 3). Unbiased and supervised classification analysis using Random Forest (RF) analysis demonstrated that the placental metabolome differentiated the control and LPS groups with an overall predictive accuracy of 87%, indicating the pronounced differences in biochemical profiles between groups (Supplementary Figure S2).

#### Acylcarnitines Are Significantly Increased in LPS Placenta

**FIGURE 4:**
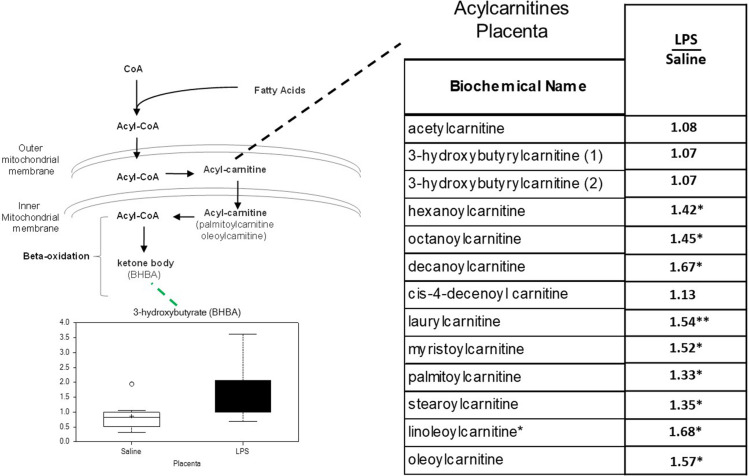
Acylcarnitine metabolism is disrupted in LPS Placenta. Relative values of differentially expressed metabolites in LPS placenta expressed as fold change of controls. ^∗^Significantly different between LPS and control placenta, *p* ≤ 0.05 and *q* ≤ 0.05. ^∗∗^Significantly different between LPS and control placenta, *p* ≤ 0.05 and *q* ≤ 0.1. Box plots of 3-hydroxybutryate in LPS (black boxes) and control (white boxes). *N* = 8 both groups.

In previous studies of human placenta from SPTB pregnancies we noted marked abnormalities in lipid metabolites, in particular acyl-carnitines, compared to placenta from term pregnancies (Elshenawy et al., 2020). Similarly, significant increases were observed in several long-chain acylcarnitines in LPS placentas. Of the 14 acylcarnitines detected, 9 were significantly elevated, and none were decreased (*p* < 0.05; Figure 4).

#### Intrauterine Inflammation Significantly Alters Glucose Utilization

**FIGURE 5:**
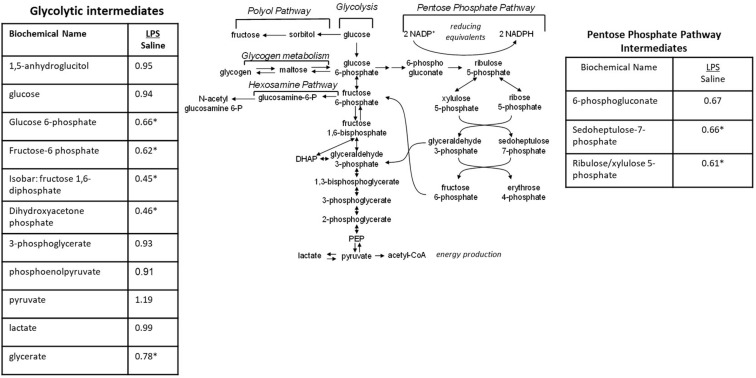
Alterations in Glucose Utilization in LPS Placenta. Relative values of differentially expressed metabolites in LPS placenta expressed as fold change of controls. ^∗^Significantly different between LPS and control placenta, *p* ≤ 0.05 and *q* ≤ 0.05. *N* = 8 both groups.

When the metabolic profiles of the LPS- and Saline-treated groups were compared, significant alterations were observed in several glucose-derived metabolites. As shown in Figure 5, the LPS-exposed animals exhibited significantly lower levels of metabolites involved in the glycolytic, pentose phosphate, and glycogen synthesis/degradation pathways. The affected pathways converge at glucose 6-phosphate (G6P) within the cell. G6P functions as an early stage component in the glycolytic and pentose phosphate pathways; it is also consumed and generated, respectively, when glycogen is synthesized and degraded.

#### Intrauterine Inflammation Perturbs Branched-Chain Amino Acid Catabolism

Significant increases were observed in several metabolites involved in branched-chain amino acid (BCAA) (leucine, isoleucine, and valine) metabolism in placenta of LPS pregnant mice. As shown in Table 4, branched-chain keto acids and hydroxycarboxcylic acids (2-hydroxy-3-methylvalerate, 3-hydroxyisobutyrate, 3-hydroxyisobutyrate, and alpha-hydroxy-3-methylvalerate) were particularly elevated. These changes are consistent with alterations in the BCAA catabolic pathway.

**TABLE 4:** Branched chain amino acids are altered in LPS placenta.

Biochemical name	Fold change	*p*-value	*q*-value
Leucine	1.04	0.3126	0.3871
N-acetylleucine	1.42*	0.0038	0.0192
Isovalerylglycine	1	0.9465	0.6335
Isovalerylcarnitine	1.3	0.0485	0.1161
beta-hydroxyisovalerate	1.43*	0.0107	0.039
beta-hydroxyisovaleroylcarnitine	0.98	0.8296	0.6113
alpha-hydroxyisovalerate	1.33	0.0598	0.1324
Methylsuccinate	1.04	0.5638	0.5213
Isoleucine	1.06	0.1286	0.2173
N-acetylisoleucine	1.13	0.1485	0.2407
2-methylbutyrylcarnitine	1.94*	5.46E-05	0.0023
Tiglylcarnitine	1	0.9681	0.6394
2-hydroxy-3-methylvalerate	1.83*	0.0005	0.0076
Ethylmalonate	1.59*	3.08E-05	0.0021
Valine	1.08	0.1021	0.1876
N-acetylvaline	1.05	0.3429	0.4056
Isobutyrylcarnitine	1.76*	0.0001	0.0038
Isobutyrylglycine	1.15	0.5306	0.5092
3-hydroxyisobutyrate	1.34*	0.0203	0.0619
alpha-hydroxyisocaproate	1.47*	0.0008	0.0091

Relative values of differentially expressed metabolites in LPS placenta expressed as fold change of controls. *Significant difference in LPS-exposed placenta compared to control, p ≤ 0.05 and q ≤ 0.1.

#### Intrauterine Inflammation Alters Carbon Flow Through the TCA Cycle

**FIGURE 6:**
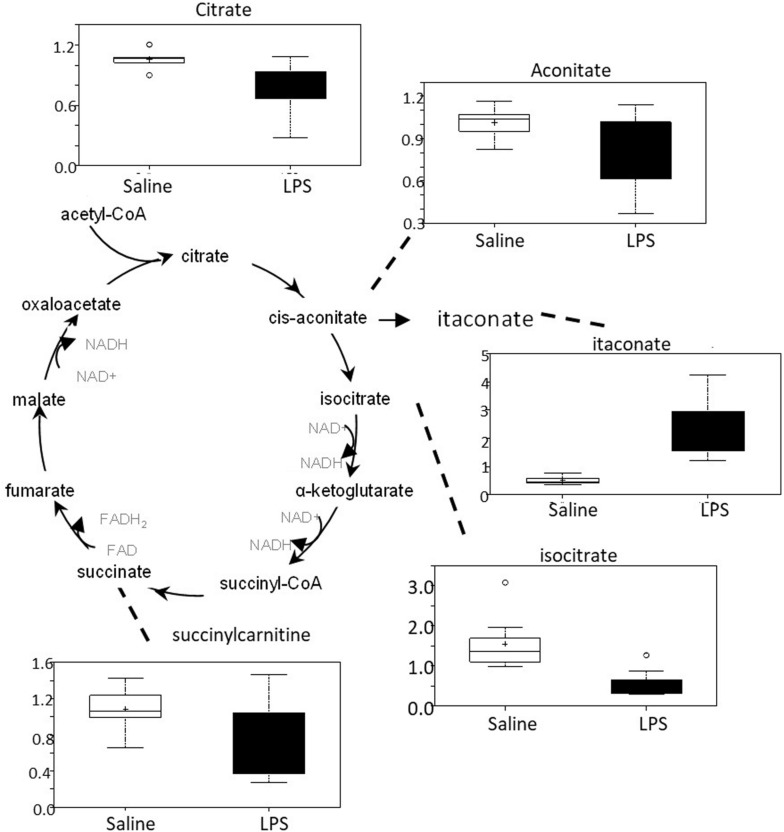
TCA cycle activity is abnormal in LPS placenta. Box plots of TCA cycle metabolites in LPS (black boxes) and control (white boxes). *N* = 8 both groups.

There was an overall decrease in several TCA cycle intermediates in LPS placenta compared to controls (citrate, aconitate, isocitrate, and succinylcarnitine) (Figure 6). These changes are consistent with alterations in carbon flow through the TCA cycle. It appears that the LPS-exposed mice shunted carbon flow in the cycle toward itaconate (methylenesuccinate) production. As shown in Figure 6, itaconate can be derived from the TCA cycle intermediate *cis*-aconitate. Itaconate inhibits isocitrate lyase, a key enzyme of the glyoxylate cycle (facilitates the conversion of acetyl-CoA to succinate). Itaconate can also inhibit glycolysis by inhibiting the formation of fructose 2,6 phosphate (which activates the glycolytic enzyme phosphofructokinase 1).

#### Purine and Pyrimidine Catabolites Accumulate in the Placentae From LPS-Exposed Dams

**FIGURE 7:**
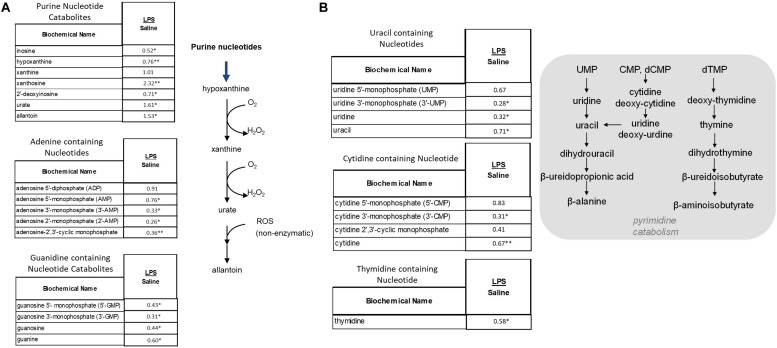
Alterations in Purine **(A)** and Pyrimidine **(B)** Metabolism in LPS Placenta. Relative values of differentially expressed metabolites in LPS placenta expressed as fold change of controls. ^∗^Significantly different between LPS and control placenta, *p* ≤ 0.05 and *q* ≤ 0.05. ^∗∗^Significantly different between LPS and control placenta, *p* ≤ 0.05 and *q* ≤ 0.1. *N* = 8 both groups.

As shown Figure 7A, these metabolic pathways showed a shift toward increased purine breakdown. Large increases were observed in hypoxanthine, xanthine, and urate (Figure 7A). LPS placentas also exhibited large decreases in adenosine and guanidine containing nucleotides. Pyrimidine catabolites trended toward lower levels in placental tissues (Figure 7B). In addition to alterations in DNA and RNA turnover in placenta, it should also be noted that these changes may correlate in part with changes in energy homeostasis as certain nucleotides (e.g., ATP) play important roles in energy metabolism.

#### Corticosterone Production Increased in the LPS-Exposed Mice

LPS-exposed mice exhibited significant increases in corticosterone (1.76-fold increase over saline, *q* < 0.005) and 11-dehydrocorticosterone (2.34-fold increase over saline, *q* < 0.005) in placental. These changes are consistent with increased corticosterone production. Corticosterone, the principal glucocorticoid produced in mice, is involved in mediating energy regulation, immune reactions, and stress responses.

#### Nicotinamide Metabolism

**FIGURE 8:**
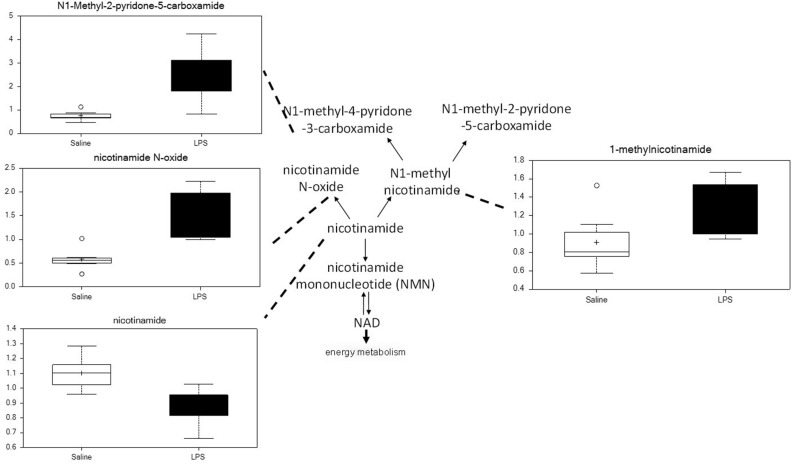
Maternal LPS increases nicotinamide degradation in placenta. Box plots of nicotinamide metabolites in LPS (black boxes) and control (white boxes). *N* = 8 both groups.

Placenta from LPS-exposed mice exhibited significant increases in several metabolites that are derived from nicotinamide. As shown in Figure 8, placenta of these animals contained higher levels of nicotinamide, nicotinamide N-oxide, 1-methylnicotinamide, and N1-methyl-2-pyridone-5-carboxyamide. These changes are consistent with increases in nicotinamide catabolism and likely correlate with the changes in energy metabolism discussed above.

#### Choline Metabolism

Choline and several choline-derived metabolites in this study were markedly altered in LPS placenta (Table 5). Choline was significantly elevated, whereas cytidine 5′-diphosphocholine and glycerophosphorylcholine (intermediates in phosphatidylcholine synthesis and degradation) were lower in LPS placenta.

**TABLE 5:** Choline-derived metabolites are altered in LPS placenta.

Biochemical name	Fold change	*p*-value	*q*-value
Choline	1.13*	0.0252	0.072
Choline phosphate	0.7*	0.0114	0.041
Cytidine 5′-diphosphocholine	0.43*	0.0269	0.0749
Glycerophosphorylcholine	0.81*	0.006	0.0254

Relative values of differentially expressed metabolites in LPS placenta expressed as fold change of controls. *Significant difference in LPS-exposed placenta compared to control, p ≤ 0.05 and q ≤ 0.1.

### Interactome Network Analysis of the Transcriptome and Metabolome in LPS-Exposed Placentas

**FIGURE 9:**
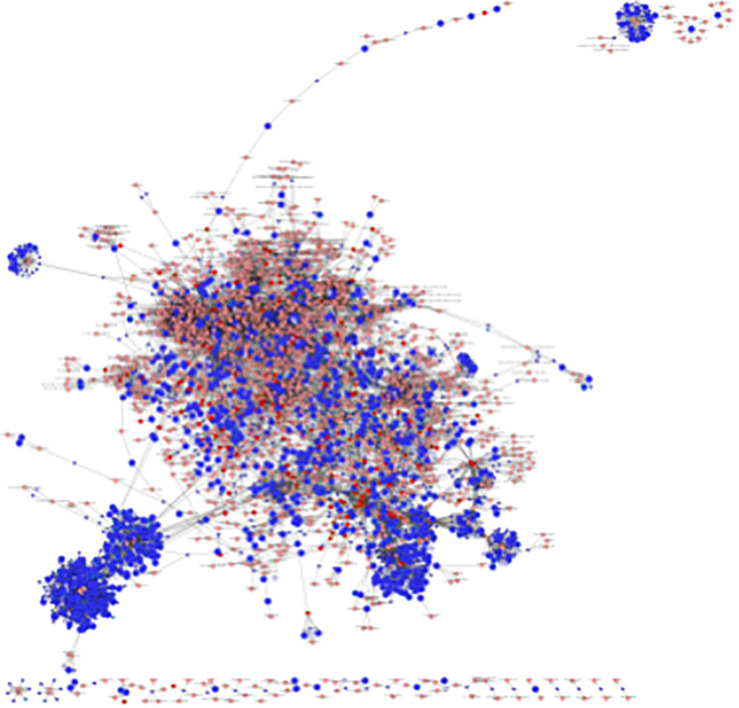
Visual representation of the interactome model. Interaction network of integrated transcriptome and metabolome was analyzed using MetScape 3.1. Dark blue squares represent differentially expressed genes in the placenta dataset; light blue squares represent inferred gene interactions; dark red squares represent significantly changed metabolites in the placenta dataset; light red squares represent inferred metabolite interactions; gray lines represent protein-protein or protein-metabolite interactions.

An interactome network model (Figure 9) integrating transcriptomic and metabolomic was generated which connected pathways via protein-protein or protein-metabolite interactions. Analysis of this interactome network demonstrated that several critical metabolic processes were altered in LPS-exposed placenta. Not surprisingly, these modules included processes associated with glycolysis, and gluconeogenesis, pentose phosphate pathway, TCA cycle, amino acid metabolism, purine, and pyrimidine metabolism, phosphatidylinositol phosphate metabolism, glycosphingolipid metabolism, glycerophospholipid metabolism, and nicotinate and nicotinamide metabolism (Table 6). The differentially expressed genes and significantly changed metabolites associated within these modules in our datasets are listed in Table 6.

**TABLE 6:**
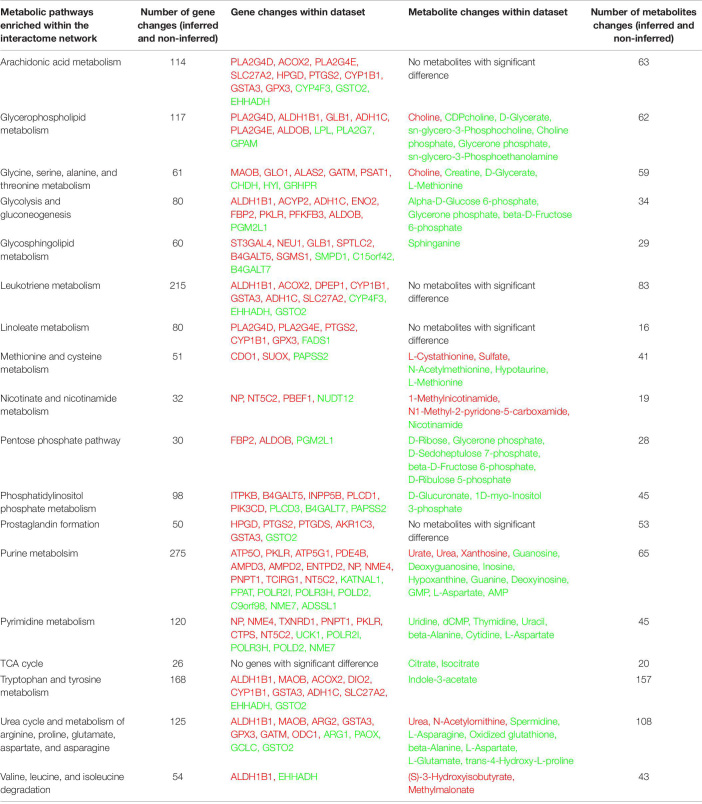
Metabolic pathways identified from the interactome network.

*Genes or metabolites shown in red were up-regulated, whereas those in green were down-regulated.*

## Discussion

Using a model of intrauterine inflammation that is similar to human pregnancies complicated by acute chorioamnionitis (Elovitz and Mrinalini, 2005; Elovitz et al., 2011; Hester et al., 2018; Brown et al., 2019), we demonstrated that intrauterine LPS injection alters the placenta metabolome and is associated with marked changes in expression of genes involved in key pathways including vascular function and reactivity, mitochondria function and nutrient sensing, glucose and lipid metabolism, and ceramide and sphingosine-1-phosphate signaling.

One of the more surprising findings in our study was the alteration of a large number of genes and pathways that regulate vascular function and reactivity including renin-angiotensin signaling (RAS) in placenta exposed to intrauterine inflammation. RAS was one of the most significantly activated pathways in LPS-exposed placenta. Renin-angiotensin signaling regulates systemic blood volume and maternal-fetal blood flow during pregnancy, and has multiple effects in vascular remodeling and reactivity. During pregnancy, estrogen up-regulates renin and angiotensinogen levels leading to increased activity for both systemic and local uteroplacental renin-angiotensin system (RAS) (Anton and Brosnihan, 2008; Clark et al., 2017). RAS activity is significantly higher in pre-eclamptic placentas (Singh et al., 2004), which is associated with hypertension, reduced maternal-fetal blood flow, limited nutrient and oxygen supply to the fetus, as well as intrauterine growth restriction (Clark et al., 2017). Furthermore, angiotensin II can directly decrease amino acid transporter activities in human placenta (Shibata et al., 2006). Thus, increased renin-angiotensin signaling activity may play a critical role in acute intrauterine inflammation-induced alterations of metabolic processes and poor fetal outcomes.

A number of other pathways regulating vascular function were also significantly altered in LPS placenta including sphingosine-1-phosphate signaling and the PI3K/AKT/PTEN signaling pathway. Spingosine-1-phosphate signaling controls vascular tone, permeability, and modulation of α_1_-adrenergic induced vasoactivity (Hla, 2003; Maceyka et al., 2012). The major components of the PI3K/AKT/PTEN signaling pathway are abundantly expressed in placenta and among their myriad functions they also regulate vascular function, including vascular tone, angiogenesis, and control of adhesion in the placenta (Yang et al., 2003; Hamada et al., 2005; Jiang and Liu, 2009). Taken together, our finding that multiple pathways regulating vascular biology were markedly altered in LPS exposed placenta suggests that one of the major complications of intrauterine inflammation is the disruption of vascular tone and reactivity in the placenta which can have profound implications for placenta function and fetal outcomes.

Another key finding was altered acylcarnitine metabolism in LPS placenta. This is very similar to our observations in human placentas from SPTB suggesting that these lipid species play a fundamental role in placental failure associated with inflammation and preterm birth. Acylcarnitines are intermediate oxidative metabolites. They consist of a carnitine moiety that facilitates its transport across the mitochondrial membrane for β-oxidation (Batchuluun et al., 2018). In general, acylcarnitines transport long-chain fatty acids into mitochondria and generate acetyl CoA and ATP. High levels are secondary to either altered fatty acid transport and/or oxidation rates. Our finding of increased levels of 3-hydroxybutyrate (BHBA) in the placenta suggests that impaired fatty oxidation rates are responsible for increased acylcarnitines in LPS placenta. BHBA is a ketone body that typically increases in concentration during ketogenic conditions and decreased β-oxidation. Indeed, in our previous studies of SPTB placenta, fatty acid oxidation rates were markedly decreased (Elshenawy et al., 2020). However, the direct function of acylcarnities in the placenta has not been well characterized. Human placenta expresses high levels of enzymes involved in fatty acid oxidation (Shekhawat et al., 2003). Acylcarnitines are thought to activate proinflammatory signaling, engage pattern recognition receptor (PRR)-associated pathways, and induce the expression of cyclooxygenase-2 leading to uncontrolled inflammation (Rutkowsky et al., 2014). Acylcarnitines can also induce mitochondrial dysfunction (Batchuluun et al., 2018). This ongoing oxidative stress and inflammation may ultimately lead to severe placental dysfunction and disruption of fetal membranes, resulting in preterm birth. Furthermore, elevated levels of free carnitine and several short-chain, medium-chain, and long-chain acylcarnitines in the circulation have been observed in adverse pregnancy complications such as preeclampsia and gestational diabetes, both of which are associated with placental dysfunction (Koster et al., 2015; Batchuluun et al., 2018).

We identified multiple additional metabolic pathways that were altered in the placenta from dams exposed to intrauterine inflammation indicating that intrauterine inflammation has broad adverse effects on placenta metabolism. Placenta from dams exposed to intrauterine inflammation exhibited significant alterations in the availability and utilization of several energy sources including glucose, branched-chain amino acids, and fatty acids. These changes were accompanied by alterations in TCA cycle intermediates, nucleotide metabolites, oxidized lipids, and steroid metabolites. Notable changes were also apparent in nicotinamide catabolites, choline metabolites, and dipeptides. Localized intrauterine inflammation appeared therefore to have major impacts on both the transfer and utilization of metabolites in the intrauterine space. Metabolites that were altered in each class may play a role in placental dysfunction through energy failure, inflammation, early senescence, and maternal-fetal intolerance.

An additional important finding was the marked changes in branched-chain amino acid (BCAA) metabolism in LPS placenta. BCAA catabolism is mediated by two major enzymes: (1) branched-chain aminotransferase (BCAT) and (2) branched-chain keto acid dehydrogenase (BCKD). BCAT is responsible for converting BCAAs to branched-chain keto acids (BCKAs) and BCKD is responsible for converting BCKAs to branched-chain acyl-CoA intermediates. The latter may be conjugated to carnitine and subsequently metabolized to either propionyl-CoA or acetyl-CoA in the mitochondria. When alterations are present in this pathway, however, branched-chain keto acids may accumulate and/or be converted to 2-hydroxycarboxylic acids (as observed in this study). The accumulation of these metabolites in this study therefore, suggest that intrauterine inflammation alters BCAA breakdown.

There was significant overlap between the transcriptome and metabolomic data. Both data sets showed abnormalities in mitochondrial function, nutrient sensing, and glucose and lipid metabolism in the placenta from dams exposed to intrauterine inflammation. Similarly, the metabolomics data showed significant alterations of many glucose-derived metabolites and lipid metabolites especially long-chain acylcarnitines, suggesting the dysregulation of glucose utilization and lipid metabolism in LPS placenta. Increased acylcarnitines in LPS placenta may further exacerbate inflammation, induce mitochondrial dysfunction, and increase oxidative stress, all of which were observed in transcriptome data by IPA analysis. Acylcarnitines can cause uncontrolled inflammation via up-regulation of cyclooxygenase-2 (Rutkowsky et al., 2014). Indeed, the expression of cyclooxygenase-2 (*Ptgs2*) was increased in LPS placenta. It was also predicted as an activated upstream regulator by IPA analysis. TCA cycle intermediates were altered in LPS-exposed mice. The formation of itaconate, a derivative from TCA cycle intermediate cis-aconitate by immunoresponsive gene 1 (Irg1), a gene that is highly expressed during infections (Michelucci et al., 2013), was significantly increased in LPS placenta. Consistent with increased itaconate, the expression of *Irg1* was increased 10-fold in LPS placenta. Accumulation of purine and pyrimidine catabolites were also found in LPS-exposed mice in our metabolomics studies, which was supported by the alteration of pathways regulating pyrimidine ribonucleotides *de novo* biosynthesis and interconversion in the transcriptome data. Furthermore, the P2Y purigenic receptor signaling pathway that mediates purines and pyrimidines signaling was activated in LPS placenta.

Integrated interactome model provides greater confidence of the signaling and pathways identified in the transcriptome and metabolome individually. Consistent with our previous findings in human SPTB placenta, alteration of lipid metabolism in LPS-exposed placentas was identified in all three analyses of transcriptome, metabolome, and interactome, which further supports that aberrant fatty acid metabolism in the placenta is highly associated with preterm birth. In addition to the changes in glucose utilization and BCAA catabolism identified in metabolome, interactome model showed that metabolism for almost all amino acids were altered in LPS-exposed placentas. Collectively, acute intrauterine inflammation leads to alterations and/or deficits in the metabolism of glucose, amino acids, lipids, purine, and pyrimidine in placenta.

A well-functioning placenta plays a crucial role in normal pregnancy. Our current study has identified alterations in novel pathways and upstream regulators that may play an important role in the maintenance of normal bioenergetic metabolism. Results from our current study in the acute intrauterine inflammation mouse model have many similarities with results from our previous metabolomics study in human placentas from SPTB (Elshenawy et al., 2020). Since inflammation is one of the major causes of SPTB in human, our findings provide new insights into the underlying mechanisms of SPTB.

## Data Availability Statement

The datasets generated for this study can be found in the NCBI GEO accession GSE151728.

## Ethics Statement

The animal study was reviewed and approved by The University of Pennsylvania Institutional Animal Care and Use Committee.

## Author Contributions

Y-CL and RS: conceptualization, writing—original draft preparation, and project administration. Y-CL, GB, AG, and ME: methodology. Y-CL: validation. ZZ: formal analysis. Y-CL, ZZ, and RS: data curation. RS: supervision, writing - review and editing. RS and ME: funding acquisition. All authors have read and agreed to the published version of the manuscript.

## Conflict of Interest

The authors declare that the research was conducted in the absence of any commercial or financial relationships that could be construed as a potential conflict of interest.

## Footnotes

1 https://github.com/alexdobin/STAR
