# Supplementary material for: Intrauterine Inflammation Alters the Transcriptome and Metabolome in Placenta
Source: Front Physiol. 2020 Nov 5;11:592689. doi: 10.3389/fphys.2020.592689 (PMC7674943; doi:10.3389/fphys.2020.592689)
Supplement: Supplementary file 1 [file Data_Sheet_1.PDF]

Supplemental Figure 1. Connected regulators of master regulators

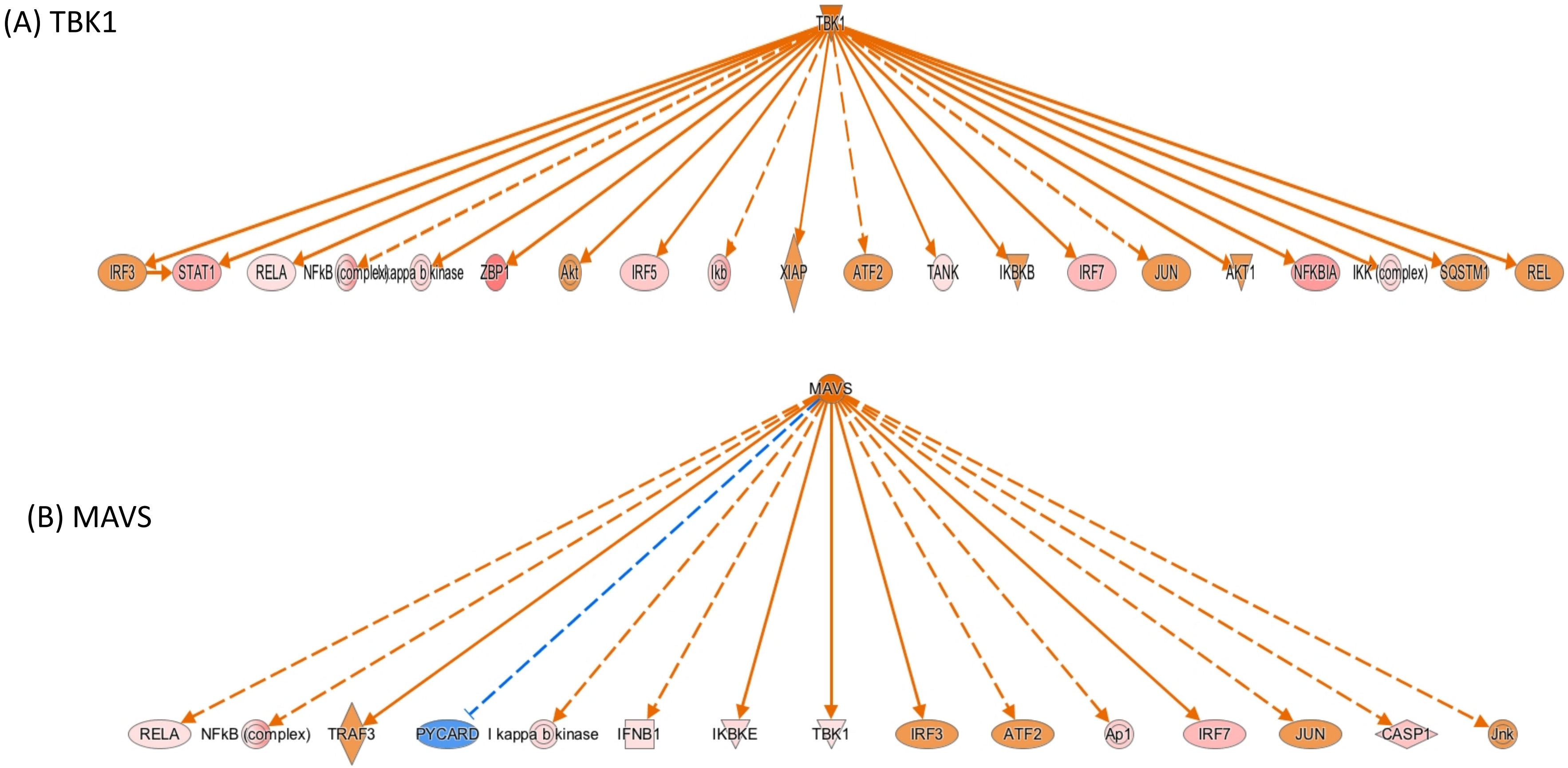

Supplemental Figure 1: Ingenuity Pathway Analysis® (IPA) annotated regulator networks connecting to master regulators. Upstream regulators connecting to master regulator TBK1 (A), MAVS (B), STIM1 (C), STAT3 (D), and STAT1 (E). Orange-filled and blue-filled shapes indicate predicted activation and inhibition, respectively; red-filled and green-filled shapes indicate increased and decreased expression, respectively; orange-red lines indicate activation; blue lines indicate inhibition; yellow lines indicate findings inconsistent with state of downstream activity.

Supplemental Figure 1. Connected regulators of master regulators

(C) STIM1

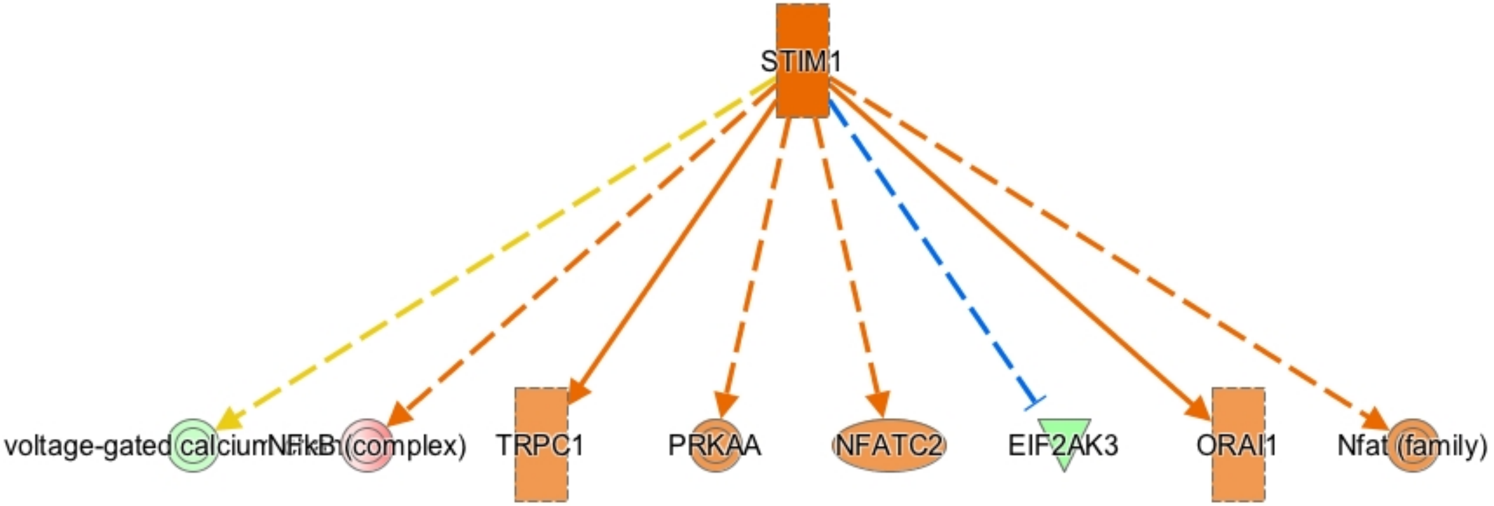

(D) STAT3

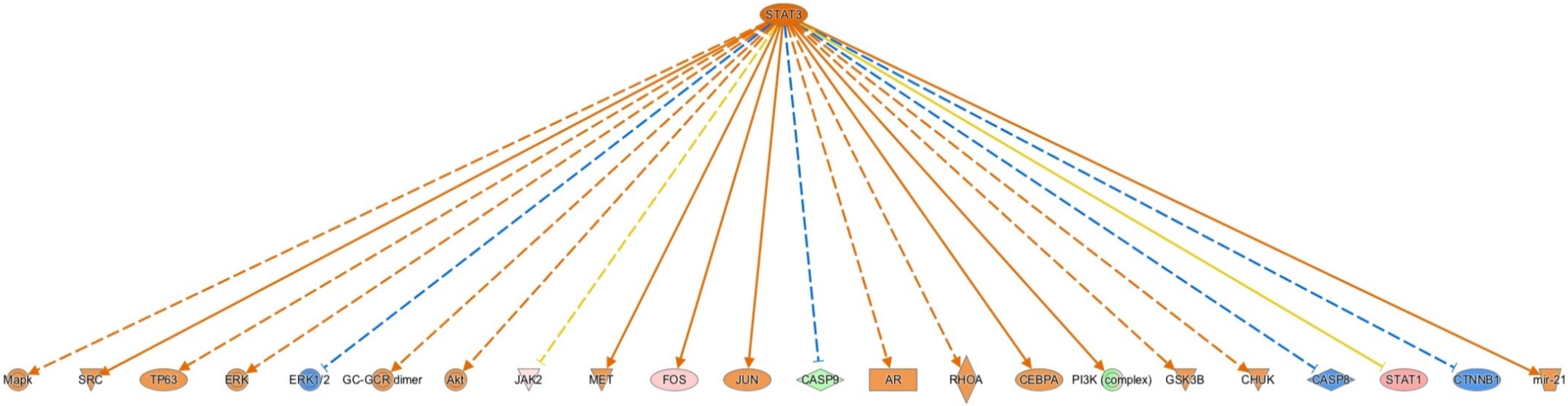

Supplemental Figure 1. Connected regulators of master regulators

(E) STAT1

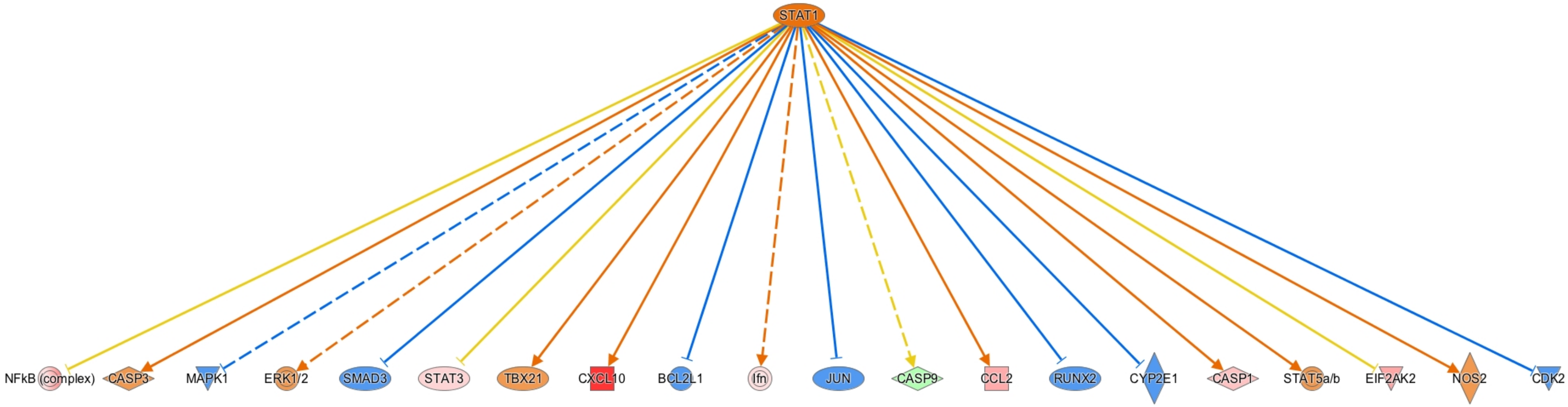

Supplemental Figure 2. Random Forest Plot

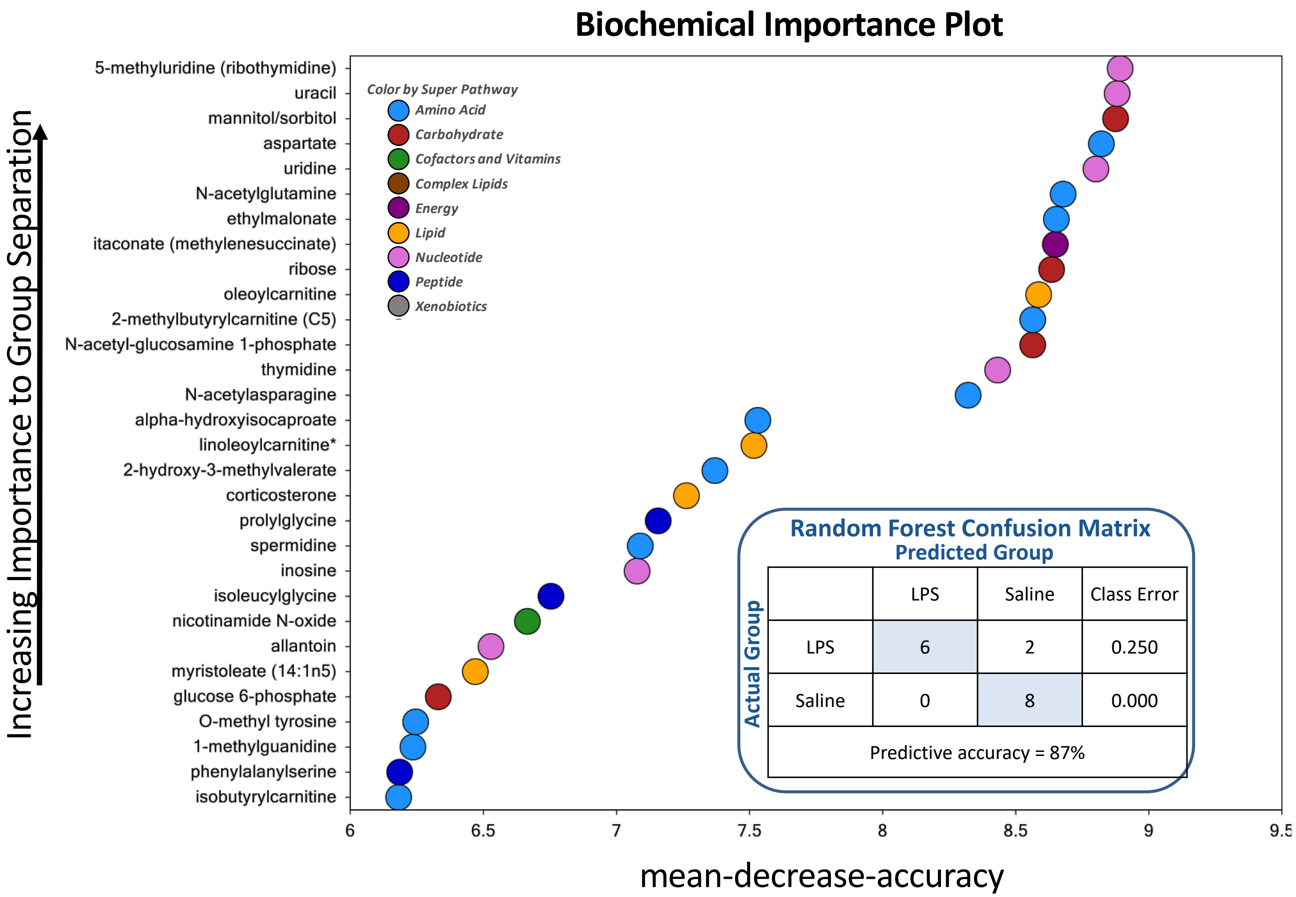

	LPS	Saline	Class Error
LPS	6	2	0.250
Saline	0	8	0.000
Predictive accuracy = 87%			

Supplemental Figure 2: Random forest classification of LPS-treated placenta samples that underwent metabolomic analysis listed by variable importance, and colored-coded by biochemical class. Random forest classification is an unsupervised analytic tool for ranking the importance of variables within a data set. The X-axis represents the mean decrease accuracy (MDA), a calculated metric of biochemical importance in characterizing the overall differences between experimental groups. A higher MDA indicates greater importance in characterizing the differences between LPS and control placentas.
